# Effects of a videoconference-based therapeutic exercise intervention on the musculoskeletal pain of eldercare workers: protocol for the ReViEEW randomized controlled trial

**DOI:** 10.1186/s12891-023-06584-7

**Published:** 2023-06-06

**Authors:** Ander Espin, Jon Irazusta, Itziar Segovia Celaya, Álvaro Mosquera Lajas, Vanesa González-Templado, Ana Rodriguez-Larrad

**Affiliations:** 1 Ageing On Research Group, Department of Physiology, University of the Basque Country (UPV/EHU), Leioa, Spain; 2 Biocruces Bizkaia Health Research Institute, Barakaldo, Spain; 3 Home Care Lab, S. Coop., Bilbao, Spain; 4 Fundación Aspaldiko, Portugalete, Spain; 5 Caser Residencial Betharram, Hondarribia, Spain

**Keywords:** Clinical trial, Study protocol, Tele-rehabilitation, Resistance exercise, Musculoskeletal disorders, Pain, Occupational health, Physical fitness, Mental health, Quality of life

## Abstract

**Background:**

Prevalence of musculoskeletal pain is high among eldercare workers, and therapeutic exercise has shown to be effective for its management. Although telerehabilitation is an increasingly used alternative for delivering therapeutic exercise, no studies have assessed synchronous group telerehabilitation interventions for the management of musculoskeletal disorders. Thus, the aim of this article is to describe the protocol of a randomized controlled trial that will assess the effects of a videoconference-based group therapeutic exercise intervention on the musculoskeletal pain of eldercare workers.

**Methods:**

This multicenter trial will randomly assign 130 eldercare workers to either a control or experimental group. Participants in the control group will not receive any intervention, and participants in the experimental group will take part in a 12-week remote supervised videoconference-based intervention, consisting of 2 weekly 45-min group sessions. Each session will include 4 sets of 6 progressive resistance exercises for the lower limbs, upper limbs and trunk, performed with bodyweight and elastic bands at moderate-high intensity. Following the 12 weeks, participants in the experimental group will be provided with material for autonomously carry on the therapeutic exercises and advised to continue performing 2 weekly sessions on their own until a 48-week follow-up. Assessments will be performed at baseline, 12 and 48 weeks. Primary outcome will be average pain intensity in the low back during the last 7 days, measured by the 0–10 Numerical Rating Scale. Secondary outcomes will include additional measures of musculoskeletal pain, psycho-affective state, work-related variables, and physical fitness.

**Discussion:**

This will be the first trial, to our knowledge, assessing whether a remote delivery of a group therapeutic exercise intervention via videoconference is effective for reducing the musculoskeletal pain, improving the psycho-affective state and physical fitness, and enhancing the work-related parameters in eldercare workers. If successful, this study will provide innovative tools for implementing effective, scalable and affordable interventions to tackle musculoskeletal disorders in the workplace. It will also highlight the utility of telehealth, and address the importance of therapeutic exercise to manage musculoskeletal pain in a critical population for the future of the aging societies as it is the eldercare workers.

**Trial registration:**

The study protocol was prospectively registered at ClinicalTrials.gov (registration number: NCT05050526) on September 20, 2021.

## Introduction

Eldercare workers are qualified professionals who provide assistance to dependent elderly people at either home or long-term facilities [1]. Demographic projections for the coming decades suggest that the demand for eldercare will at least double by 2050 [2], which makes these professionals a cornerstone for facing the challenge of aging. Taking care of people with moderate or severe disability is a physically demanding task, involving lifting, transferring and other care activities that might lead to an overload of musculoskeletal tissues [3]. Several studies have shown that prevalence of pain is high among eldercare workers [4, 5], with 88% of these professionals reporting at least one body part with work-related musculoskeletal symptoms [6]. Besides, prospective studies with large samples have found that a higher intensity [7] and frequency [8] of pain, as well as a higher number of pain locations [9], are significant risk factors for long-term sickness absence in eldercare workers. Moreover, the presence of pain-related disability and a longer pain duration predict the risk of dropout or job turnover from the eldercare sector [10]. In eldercare workers, musculoskeletal pain is often accompanied by mental health disorders [11–13], which can be exacerbated by the high psychological demands of the profession [14–16].

Physical activity and therapeutic exercise interventions have been shown to be effective in reducing musculoskeletal pain in the general adult population [17], and are included as a first-line treatment in all high-quality clinical practice guidelines with the most up-to-date evidence [18]. Although the biological mechanisms leading to exercise-induced hypoalgesia are not yet fully understood, it seems that the activation of the endogenous opioid system during exercise plays a key role [19]. However, it has been suggested that the endocannabinoid, serotonergic, immune and autonomic nervous systems may also be involved, and there are several psychosocial factors that could influence the exercise modulation of pain [19]. From a biomechanical point of view, improvements in the structure and function of the musculoskeletal system, especially muscle strength, could explain the pain reduction induced by exercise [20].

Telerehabilitation is an increasingly used alternative for remotely delivering health services using telecommunications technologies [21, 22]. Although previous studies reported some positive effects on pain and other health-related outcomes, a recent review stated that it is imperative to conduct high quality clinical trials in order to identify effective telerehabilitation interventions [21]. To the knowledge of the authors, the great majority of internet-based interventions have consisted of websites with content for autonomous consultation, or individual home-videoconference sessions [21–25]. There are two positive experiences showing that synchronous supervised sessions are a feasible way to present group therapeutic exercise interventions in people with chronic obstructive pulmonary disease [26] and older adults [27], but no study has been addressed to assess their effectiveness on musculoskeletal disorders yet. Synchronous supervision and group dynamic could be important for designing exercise programs, as both features are related to higher participant adherence, what may therefore lead to a higher effectiveness [28, 29]. In addition, it could also allow for greater intervention safety, since participants can be continuously monitored for correct execution during exercising.

Thus, a study protocol for a randomized controlled trial was designed with the aim of assessing the effects of a videoconference-based group therapeutic exercise intervention in the medium and long term on the musculoskeletal pain of eldercare workers. Secondary outcomes will include measures related to the psycho-affective state, work-related variables and physical fitness.

## Methods

### Study design

**Fig. 1:**
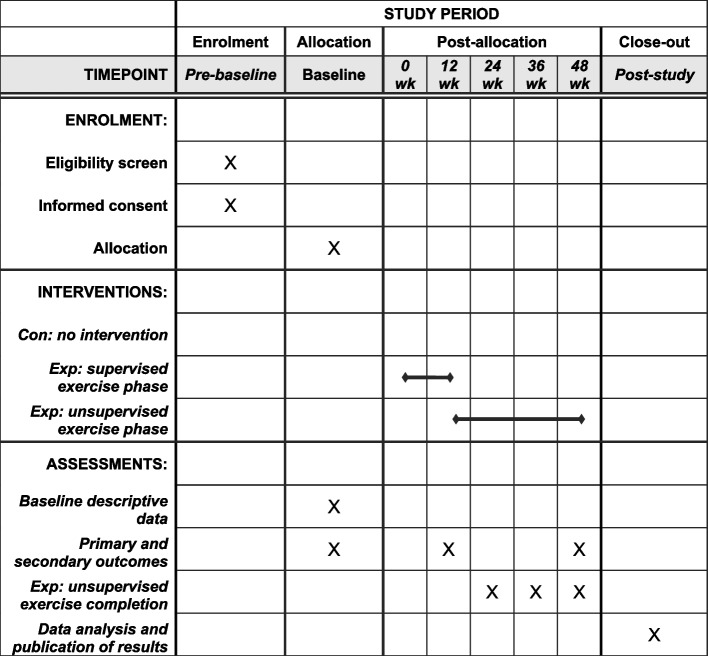
SPIRIT flow diagram for the schedule of enrollment, interventions, and assessments. *Legend*: Con: control group; Exp: experimental group; wk: week

A parallel-assignment, multicenter randomized controlled trial will be carried out. Participants will be recruited from institutions providing eldercare services at home or in long-term facilities. In each of the institutions, and following baseline measurements, participants will be randomly assigned (1:1 ratio) through sealed opaque envelopes to either a control or experimental group by a coin-tossing sequence generation. Assessments of primary and secondary outcomes will be conducted at baseline and at 12 weeks (post-supervised phase) and 48 weeks (post-unsupervised phase) from the beginning of the intervention (Fig. 1). Outcome assessors and data analysts will be blinded to group allocation. Because of the nature of the study, blinding of the participants and the professional supervising the sessions is not possible. The study was designed, and the results will be reported according to the SPIRIT statement [30] and CONSORT guidelines for trials of nonpharmacologic treatment interventions [31].

### Inclusion and exclusion criteria

Subjects will be considered eligible for the study if they meet all the following criteria: (a) are formal eldercare workers from eldercare institutions, (b) are ≥ 18 years of age, (c) have ≥ 3 months of experience in the profession, and (d) have an employment contract until at least the date of study completion. Participants will be excluded if (a) they are pregnant or (b) their participation is considered contraindicated according to the American College of Sports Medicine’s exercise preparticipation health screening guidelines [32].

### Control group

Participants in the control group will not receive any intervention and will be instructed to continue with their usual lifestyle.

### Experimental group

#### Supervised phase

**Fig. 2:**
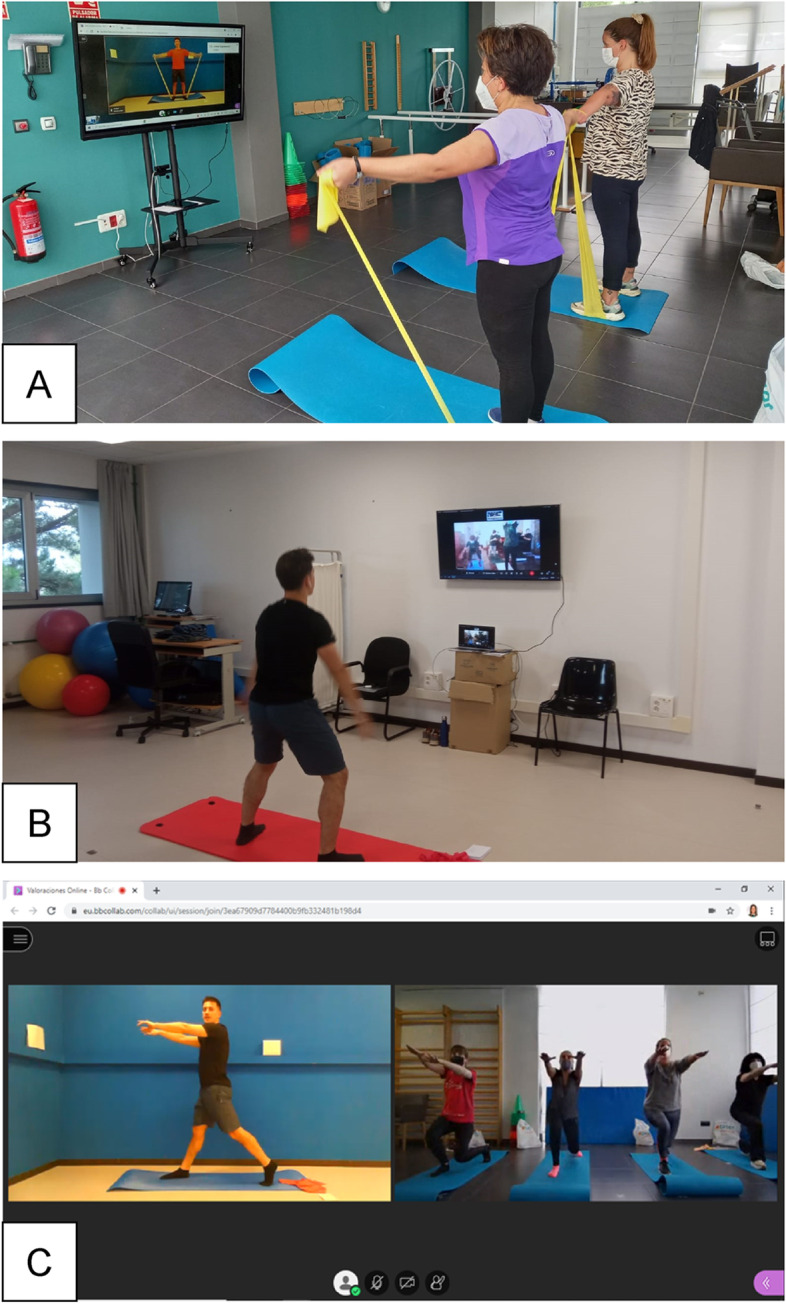
Setting up of the videoconference-supervised exercise sessions. *Legend*: **A** workplace setting in a participating nursing home; **B** setting of the professional supervising the session; **C** screenshot of a videoconference session

Participants in the experimental group will take part in a 12-week exercise intervention, consisting of two videoconference-supervised sessions per week of 45 min each. A minimum interval of 48 h will be ensured between sessions. The sessions will be carried out in groups of a maximum of 10 participants, implemented in the workplace but outside of working hours, and remotely supervised in real-time by a professional with previous experience in delivering group exercise sessions. Real-time videoconference platforms such as Webex (Cisco Systems, Milpitas, USA) will be used, and audio and video will be continuously shared between participants and trainer to allow complete bidirectional feedback (Fig. 2).

**Fig. 3:**
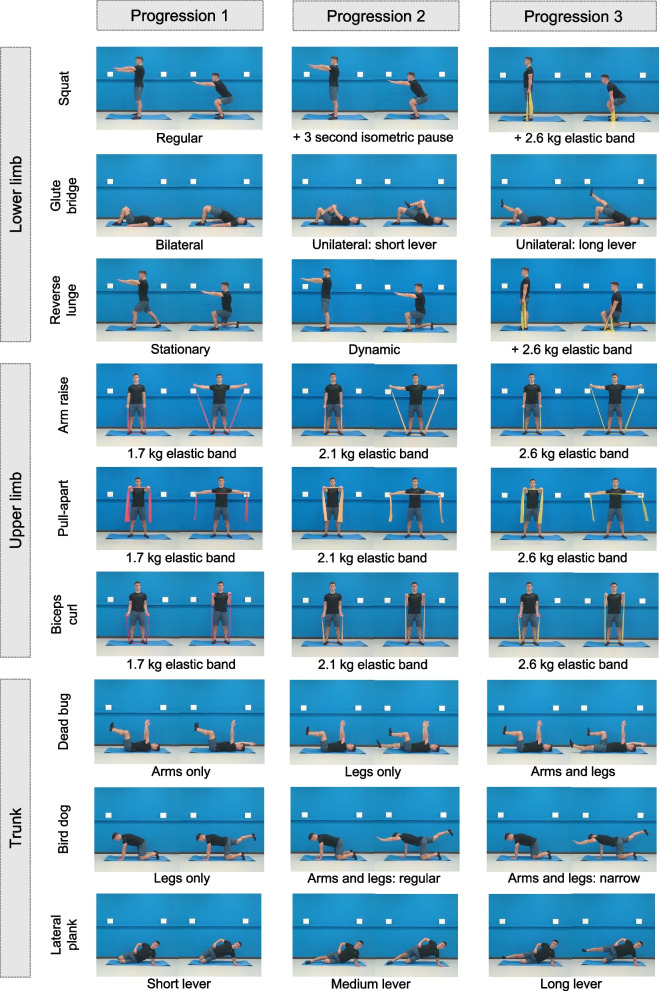
Resistance exercises performed in the program

**Fig. 4:**
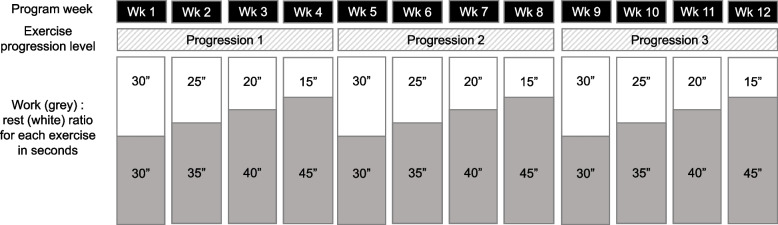
Progression of the exercises during the 12 weeks of intervention

The technical content of the program is based on a literature review, authors’ expertise and field experience, as well as on the results of a previous pilot trial that we carried out between January and March 2020 with 20 eldercare workers [33], that allowed us to define the exercises, intensities, and feasibility of a program addressed for reducing the musculoskeletal pain in this population. Intervention details are reported based on the Template for Intervention Description and Replication (TIDieR) Checklist [34]. Sessions will start with a warm-up (5–10 min), including joint mobility and aerobic activation exercises focused on increasing heart rate. The main part of the session will consist of resistance exercises (30 min) performed at moderate-high intensity. In total, 9 exercises will be performed throughout the program (Fig. 3). In each session, 4 sets of 6 resistance exercises will be performed, with a 2-min active rest (dynamic stretching and breathing exercises) between sets. Exercises will be systematically varied between sessions so that each of them is evenly performed during the program. In each set, exercises for the different major muscle groups will be alternated in a circuit manner (e.g., lower limb, upper limb, trunk, lower limb, upper limb, trunk) (Table 1). Unilateral exercises will be alternated between sets so that both sides of the body are evenly worked in each session. Exercises will be performed with minimal equipment, combining body-weight and 2-m-long elastic band exercises. Three progression levels will be set for each of the exercises: progression 1 (weeks 1–4), progression 2 (weeks 5–8) and progression 3 (weeks 9–12) (Fig. 3). All participants will start in progression 1, and transition to a subsequent progression will only be allowed if the participant completes ≥ 4 sessions in the previous progression level. Progression levels are achieved by modifying the exercise technique (e.g., increasing the force lever) or utilizing elastic bands of different resistances (1.7, 2.1 and 2.6 kg at 100% elongation for progressions 1, 2 and 3, respectively) (Fig. 3). One minute will be dedicated to the completion of each exercise (including work and rest times), and within each progression level, the work:rest ratio will augment from 30:30 to 45:15 s, thus adding 5 s of work and reducing 5 s of rest each week (Fig. 4). Participants will be asked and monitored to work at a rate of perceived exertion between 3 (moderate) and 5 (strong) on the Borg's CR-10 scale [35] and not to reach failure in any of the exercises. If any of the exercises cause intolerable pain, the 4-stage exercise adjustment model proposed by Jakobsen et al. [36] will be used: (1) reduce loading intensity (e.g., returning to a previous progression level or even performing the exercise without external resistance), (2) reduce movement velocity, (3) reduce range of motion, and (4) interrupt exercise. If an exercise needs to be interrupted, it will be replaced by a pain-free exercise focused on the same muscle group. Sessions will finish with a cool-down (5–10 min), including static stretching and breathing/relaxing exercises. Daily attendance will be recorded by the professional who supervises the sessions. This professional will also collect information on the completion and intensity of each participant's training in each session. Adherence will be reported as the percentage of sessions in which participants performed the planned training regarding completion and intensity (i.e., 24 sessions = 100% of adherence) [37].

**Table 1:** Example of the scheduling of the intervention for the 7^th^ week

Objective	Session 1	Session 2
**Warm-up** (5–10 min)	Joint mobility	Joint mobility
	Aerobic activation	Aerobic activation
**Resistance training** (30 min) 4 sets with 2-min active rest between sets	1. Squat (+ 3 s isometric pause) 40’’	1. Pull-apart (2.1 kg elastic band) 40’’
	*Rest 20’’*	*Rest 20’’*
	2. Arm raise (2.1 kg elastic band) 40’’	2. Bird dog (arms and legs: regular) 40’’
	*Rest 20’’*	*Rest 20’’*
	3. Dead bug (legs only) 40’’	3. Glute bridge (unilateral: short lever) 40’’
	*Rest 20’’*	*Rest 20’’*
	4. Glute bridge (unilateral: short lever) 40’’	4. Biceps curl (2.1 kg elastic band) 40’’
	*Rest 20’’*	*Rest 20’’*
	5. Pull-apart (2.1 kg elastic band) 40’’	5. Lateral plank (medium lever) 40’’
	*Rest 20’’*	*Rest 20’’*
	6. Bird dog (arms and legs: regular) 40’’	6. Reverse lunge (dynamic) 40’’
**Cool-down** (5–10 min)	Static stretching	Static stretching
	Breathing/relaxing exercises	Breathing/relaxing exercises

#### Unsupervised phase

When the 12-week supervised sessions are finished, participants in the experimental group will be encouraged to continue performing 2 weekly sessions on their own until the 48-week follow-up. To facilitate this autonomous training, we will create supportive material for the sessions that will include two 45-min recordings with the equivalent content to a session planned for the 12^th^ week, explanatory videos of the 9 exercises performed in the supervised program and their corresponding progressions, as well as written instructions about how to perform the exercises. To record completion in this unsupervised phase, participants will be asked, every 12 weeks and until the 48-week assessments (Fig. 1), the following questions: (a) in the last 12 weeks, in how many weeks did you perform the exercises? (0–12) and (b) on average, in each of those weeks in which you performed the exercises, on how many days did you perform them? (0–7). The result of the multiplication of both answers will be considered the number of performed unsupervised sessions. In this case, self-reported completion will be calculated as a percentage of performed sessions (i.e., 72 sessions = 100% completion in the unsupervised phase).

### Outcomes

#### Baseline descriptive data

Participants will report by a self-administered written questionnaire the following descriptive data at baseline: date of birth, sex (male/female), height (cm), mass (kg), marital status (single/married/divorced/widower), educational level (primary/secondary/tertiary education), number of children (n), children cohabiting at home (no/yes), care for dependent people outside the work environment (no/yes), working hours (hours/week), experience in the profession (years), type of work shift (rotative/fixed), night shift (no/yes), alcohol consumption (never/monthly or less/2–4 times a month/2–3 times a week/ ≥ 4 times a week) [38], tobacco consumption (daily/less than daily/not at all) [39], recreational physical activity (1–8) [40], and regular resistance-exercise training (no/yes).

#### Primary outcome

The primary outcome will be average pain intensity in the low back during the last 7 days, measured by the 0–10 Numerical Rating Scale (NRS) [41] ranging from 0 (complete absence of pain) to 10 (worst imaginable pain) (Table 2).

**Table 2:** Detailed description of the outcome measures that will be assessed in the trial

Questionnaire/Test	Functions/Parameters	Description
**Musculoskeletal pain**^**a**^		
0–10 Numerical Rating Scale (NRS) for pain [41]	Pain intensity (average and worst)	Intensity during last 7 days (0–10)
Ad hoc question	Pain frequency	Days in pain during last 7 days (0–7)
Ad hoc question	Pain interference	Days in which pain negatively interferes with work during the last 7 days (0–7)
Ad hoc question	Pain medication consumption	Days of analgesic medication consumption during last 7 days (0–7)
**Psycho-affective state**		
Subjective Happiness Scale [42]	Happiness	4 items measuring perceived current happiness (1–7)
Goldberg Anxiety and Depression Scale [43]	Anxiety and depression	9 items measuring anxious (0–9) and 9 items measuring depressive (0–9) symptoms during last month
Maslach Burnout Inventory (MBI) [44]	Burnout	9, 5 and 8 items measuring frequency of feelings related to emotional exhaustion (0–54), depersonalization (0–30) and personal accomplishment (0–48), respectively
Single-Item Sleep Quality Scale [45]	Sleep quality	Single item measuring sleep quality during last 7 days (0–10)
Ad hoc question	Hypnotic/anxiolytic medication consumption	Days of hypnotic/anxiolytic medication consumption during last 7 days (0–7)
EuroQol-5D 0–100 Health State Scale [46]	Quality of life	Single item measuring self-perceived current health state (0–100)
**Work-related variables**		
Work Ability Score (WAS) [47]	Work ability	Single item measuring self-perceived current work ability (0–10)
World Health Organization Health and Work Performance Questionnaire (HPQ) [48]	Work performance	Single item measuring self-perceived work performance during last 7 days (0–10)
Borg’s CR-10 Scale [49]	Physical exertion at work	Single item measuring self-perceived physical exertion at work during last 7 days (0–10)
Institution’s registry and self-reported	Work absenteeism	Presence of absenteeism (yes/no), days of absence (n) and reason during last year
**Physical fitness**		
5-repetition sit to stand test (5RSTS) [50]	Lower extremity muscle performance	Time to stand up from and sit down on a chair 5 times, mean of two attempts (seconds)
Kneeling push-up test (KPU) [51]	Upper body muscle performance	Maximum number of kneeling push-ups (repetitions)
Shirado-Ito trunk flexor endurance test (SIF) [52]	Trunk muscle performance	Maximum time in a defined trunk flexion position (seconds)

^a^Pain intensity, frequency and interference will be collected separately in 4 body locations: the low back, neck, shoulders, and hands/wrists. Average pain intensity in the low back will be the primary outcome measure

#### Secondary outcomes

A detailed description of all the secondary outcomes is shown in Table 2. They will include validated questionnaires and tests for evaluating: (a) musculoskeletal pain [41] (intensity, frequency, and interference) of the low back, neck, shoulders and hands/wrists; (b) psycho-affective state [42–46] (subjective happiness, anxiety and depression, burnout, sleep quality, and quality of life) which will be collected by a self-administered written questionnaire; (c) work-related variables [47–49] (work ability, performance, physical exertion, and absenteeism) which will be obtained by a self-administered written questionnaire and from the official registry of the participating eldercare institution; and (d) physical fitness (trunk, lower and upper limbs muscle performance), which will be evaluated by a battery of physical performance tests [50–52] previously validated by our research group to be carried out remotely by real-time videoconference [53]. The participant's self-reported days of medication consumption of analgesics and hypnotics/anxiolytics during the last 7 days at each assessment point will also be recorded.

### Adverse events

Adverse events occurring during the supervised exercise sessions will be collected by the professional supervising the sessions and divided into 2 types: a) technical (connection and/or operation problems with the videoconferencing system) and b) participant safety-related (pain, discomfort, or any other health-related problem appearing during the session). Adverse events will also be classified as minor (those that slightly hinder the development of the exercise session) and major (those that prevent the development of the exercise session).

### Sample size calculation

The sample size was calculated to detect a significant change in low back pain that could be relevant in terms of absenteeism from work [54]. Taking into account the average low back pain intensity of 5.0 ± 2.6 in the 0–10 NRS for pain observed in previous studies carried out by our research group in eldercare workers [55] and accepting an alpha error of 0.05 and a beta error of 0.20 in a bilateral contrast, 108 participants are necessary to detect a difference equal to or greater than 1 unit. The sample size has been increased by 20% due to expected dropouts. Therefore, the required sample will be 130 participants (65 in the control group and 65 in the experimental group).

### Statistical analysis plan

IBM SPSS Statistics 27 statistical software package (SPSS, Inc., Chicago, IL) will be used for data analysis. Normality of distribution will be checked using the Kolmogorov‒Smirnov test, and non-normally distributed variables will be square-root transformed for statistical analysis. Continuous and categorical data will be reported as mean (standard deviation) and frequency (percentage), respectively. The primary analysis will be based on intention-to-treat, including data from all participants regardless of adherence to the intervention. Additionally, a per-protocol analysis will be performed, including only data from participants with ≥ 50% adherence. Finally, a post-hoc subgroup analysis will be performed to assess the effects of the intervention on low back pain outcomes separately in participants with (≥ 1 in average 0–10 NRS) and without (< 1 in average 0–10 NRS) low back pain at baseline. Between-group comparisons at baseline will be performed with the independent samples T and Chi-squared tests for continuous and categorical variables, respectively. For continuous variables, intervention effects will be analyzed with a group-by-time ANCOVA including baseline measurements as covariates. This ANCOVA will be performed separately to assess intervention effects in two time periods: 0 vs 12 weeks and 0 vs 48 weeks. Within-group changes in each time period will be performed with the paired samples T test. Effect size will be estimated by partial eta squared (η^2^). Values for η^2^of 0.02, 0.13, and 0.26 will be considered small, medium and large, respectively [56]. For categorical variables, intervention effects will be analyzed with McNemar’s test. The level of statistical significance will be set at *p* < 0.05.

### Trial status

The trial is currently ongoing, with the first participants taking part in the intervention. Recruitment is still active and will cease when the required sample size is achieved.

### Ethics

All workers from the participating eldercare institutions will receive oral and written information about the study, including objectives, assessments and intervention details. After fully understanding the study, volunteers who meet the selection criteria will sign an informed written consent form before enrolling in the study. The study protocol was approved by the Ethics Committee for Research Involving Human Beings of the University of the Basque Country (M10/2019/200MR2), was prospectively registered in ClinicalTrials.gov (NCT05050526), and complies with the Declaration of Helsinki.

## Discussion

This manuscript describes the design of the first, to our knowledge, randomized controlled trial that will assess the effects of a videoconference-based exercise intervention on the musculoskeletal pain of eldercare workers in the medium and long term. The therapeutic exercise program is evidence-based, and has been well accepted in terms of modality, intensity and frequency, and considered useful by the eldercare population in a previous pilot study.

We have prioritized a design with simple exercises that allows clear instruction and easy execution, and that guarantees attainable remote supervision by videoconference. The intervention is also carried out with little material and at a low cost, which, if proven effective, might facilitate scaling it to different settings and populations. Moreover, videoconference-based delivery is compatible with situations in which interpersonal physical distancing is needed. Consequently, this study will provide scientific support to implement therapeutic exercise interventions in the workplace, providing innovative telehealth tools for the prevention and treatment of musculoskeletal pain.

Besides, it is widely accepted that increased wellbeing of eldercare workers could lead to a higher quality of care and, with that, a better state of health of the elderly individuals in need of assistance. In this regard, a previous study on informal caregivers found that a worse health status of the caregiver increased the risk of hospitalization of the elderly person they cared for [57]. In addition, prior studies analyzing the effects of face-to-face exercise interventions in eldercare workers reported improvements in work ability [58] and productivity [59], as well as reductions in lost work days [60] and costs of sickness absence [61]. Overall, this study could contribute to the development of more sustainable systems for long-term care, which is a global challenge included among the strategic objectives of the World Health Organization [62].

Some of the strengths of this study are its randomized controlled design, as well as its proper sample size calculation. In addition, methodological details have been thoroughly described, thus ensuring replicability. Besides, the unrestrictive selection criteria will allow the great majority of eldercare workers to participate, giving the study a pragmatic nature that allows it to be highly applicable to what would happen in a real work environment. Finally, the long-term effects of the intervention will be measured with an additional 48-week follow-up.

However, some limitations should be acknowledged. For example, the study might not be powered enough to assess the effects on the secondary outcomes, so it would probably be necessary to carry out new studies to establish reliable conclusions regarding those variables. In addition, compliance during the unsupervised phase of the study is self-reported. Lastly, due to the specificity of the sample in our study, the results may not be directly applicable to other professionals with high rates of musculoskeletal pain.

In conclusion, this study will assess the effectiveness of a videoconference-based therapeutic exercise program that, if successful, will allow to implement effective, scalable and affordable interventions to tackle musculoskeletal disorders in a critical population for the future of the aging societies as it is the eldercare workers.

## Data Availability

Not applicable.
